# Prevalence and Quality of Life of Slovenian Children with Primary Nocturnal Enuresis

**DOI:** 10.1155/2012/509012

**Published:** 2012-08-14

**Authors:** Katja Karničnik, Alenka Koren, Nastja Kos, Nataša Marčun Varda

**Affiliations:** Department of Paediatrics, University Medical Centre Maribor, Ljubljanska ulica 5, 2000 Maribor, Slovenia

## Abstract

*Aim*. To get epidemiologic data about primary nocturnal enuresis (PNE) and its influence on the quality of life of Slovenian children and adolescents and to find out the knowledge about the disorder among school population. *Methods*. Prospective epidemiologic study was performed in Slovenia in 2011 and supported with two questionnaires. The first questionnaire was distributed among primary school population that included 1248 children. The second questionnaire included 44 children, who have been treated for PNE in Nephrology Unit of our Department of Paediatrics. *Results*. PNE was diagnosed in 12.4% of children, in 11.8% of girls and in 13.0% of boys. There was evident linkage between the appearance of PNE in children and their relatives. The study showed that PNE influences the quality of life in less than half of the investigated children. Disorder restricts them mostly in relations with coevals. Knowledge about PNE among children in elementary schools was found to be insufficient. *Conclusion*. We found out that the prevalence of PNE in Slovenia is comparable to prevalence in other countries. More than half of children questioned in a survey think that PNE does not affect their lives significantly. General lack of knowledge about PNE is still a problem.

## 1. Introduction

Primary nocturnal enuresis (PNE) is defined as involuntary bladder voiding at night of a child who has never been dry for more than six months and is older than five years [1]. About 15 to 20% of five-year-old children are bedwetters with variations in frequency of bedwetting. It is also present in 5% of ten-year-old children and 1% of teenagers [2]. Some studies show that boys wet their bed more often than girls, but other studies contradict them [3].

The main pathophysiological mechanisms of PNE include decreased functional bladder capacity, increased night-time urine secretion (reduction in antidiuretic hormone secretion), and impaired wakening from sleep at filled bladder [4].

We try to suppress the disorder with general measures until the age of six.Children who are older than six years are treated with bedwetting alarm or desmopressin [5]. Sometimes some other medications like anticholinergics are used [6].

PNE can have negative influence on everyday activities, family vacations, and on child's wishes and capabilities to leave home with friends and family [7]. These children are known to be sadder, socially distant, anxious, unhappy, or even depressed [8]. They often face fear and spend most of their time in society under stress, because they are ashamed and try to cover up this disorder [9]. It is possible that the important cause for lower life quality of enuretics lays in worse sleep quality [10]. Despite the fact that most of parents accept PNE, their expectation about self-control and responsibility increase with child's growing up [11]. Additionally, study made by Esposito et al. showed that enuretics have more educational problems than healthy children; the biggest problem identified was being unable to maintain concentration [12].

The aim of our study was to get epidemiologic data about PNE and its influence on the quality of life of Slovenian children and adolescents. In addition, we wanted to determine the knowledge about PNE in general population of children.

## 2. Subjects and Methods

The research was carried out in primary schools and in the Nephrology Unit, Department of Paediatrics, Maribor, from January to September 2011. Two anonymous questionnaires were used for gathering data. In order to include the entire primary school population, we interviewed children from 6 to 15 years. Our work was divided in two parts.

The first questionnaire was completed by 1248 children, representing about 1% of all children in primary schools in Slovenia. Children from five primary schools from different parts of Slovenia participated in this part. This questionnaire was distributed among all pupils in selected schools. Children aged 6 to 10 years answered the questionnaire at home with help of their parents, and older completed it at school. The questionnaire was divided in three parts. In the first part, all children answered basic questions about their lives and health, the second part was dedicated to children with enuresis, and the third part to children who do not have such problems. The third part was especially designed to spread knowledge about enuresis among children. With this first questionnaire we wanted to get information about prevalence of enuresis among Slovenian children, information about their knowledge about this disorder and their attitude towards enuretics. We also wondered whether there was a difference in general health between children who wet their bed and those who do not.

The purpose of the second questionnaire was to evaluate the quality of life of enuretics. The questionnaires were sent to children who are treated for PNE in Nephrology Unit, Department of Paediatrics, Maribor. We received answers from 44 enuretics. This questionnaire was composed of two parts. In the first part, children answered general questions about their life and problems, in the second part we wanted to evaluate the quality of their lives. Parameters included common state of health, comprehension of themselves, free time, relationships in family, relationships with friends, and school performance. On each given group of statements, children evaluated the impact of the disorder on their lives.

Questionnaires were studied with SPSS statistic program for Windows environment 17.0.

Variables were analyzed using Pearson*'*s *χ* ^2^. Values of *P* < 0.05 were considered statistically significant.

The study was approved by the Committee for Medical Ethics, University Medical Centre Maribor (provision number 9/11).

## 3. Results

Children were between 6 and 15 years old, with average age of 9.9 years (SD ± 2.8 years). Among them, there were 642 (51.4%) girls and 569 (45.6%) boys, and in 37 (3%) we did not get defined answers. Prevalence of enuresis among children in elementary schools was found to be 12.4%. Children with enuresis were also aged between 6 and 15 years, the average age was 8.1 years (SD ± 2.7 years).

**Figure 1:**
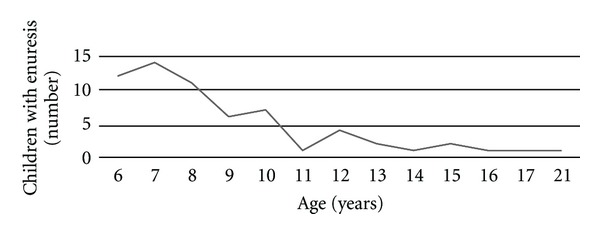
Distribution of enuretic children according to their age.

The second questionnaire was answered by enuretics aged between 6 and 21 years (average age 9.0 years, SD ± 3.7). 61% of children included were between six and eight years old. We received 19 (43.2%) replies from girls and 23 (52.3%) replies from boys. 63 children who still wet their bed were identified in both questionnaires; their age distribution is shown in Figure 1.

There were 18 parents of enuretics that were bedwetters (11.7%) (other parents only in 2.2% (*χ* ^2^ = 28.72, *P* = 0.0)). Siblings have or had the same problems in 10.4% (others only in 3.6% (*χ* ^2^ = 36.67, *P* = 0.0)) and grandparents in 4.5% (others in 1.1% (*χ* ^2^ = 2.0, *P* = 0.157)). Analysis of the second questionnaire showed similar results. 29.6% (*χ* ^2^ = 16.07, *P* = 0.0) parents of enuretics had the same problem, their grandparents in 6.8% (*χ* ^2^ = 10.71, *P* = 0.001) and their siblings in 4.6% (*χ* ^2^ = 49.28, *P* = 0.0).

**Figure 2:**
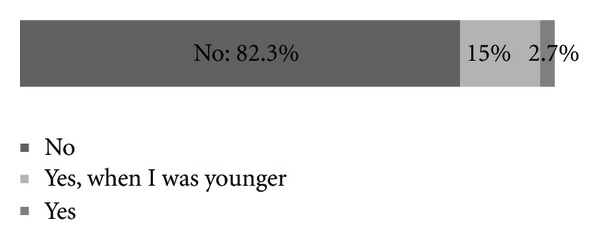
Treatment of patients with primary nocturnal enuresis (Do you receive treatment for bedwetting?).

**Figure 3:**
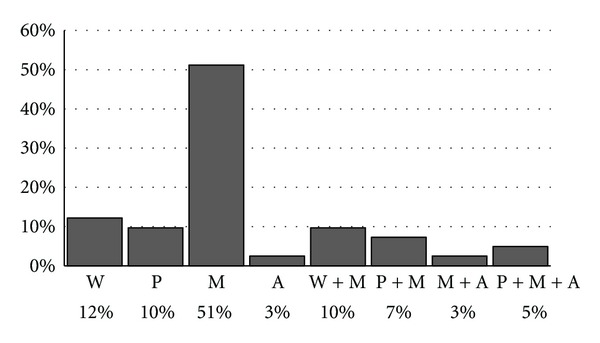
Treatment of patients with primary nocturnal enuresis (Do you know how enuresis is treated?) Abbreviations: W: waking up, P: psychologist, M: medicines, A: bedwetting alarm.

**Figure 4:**
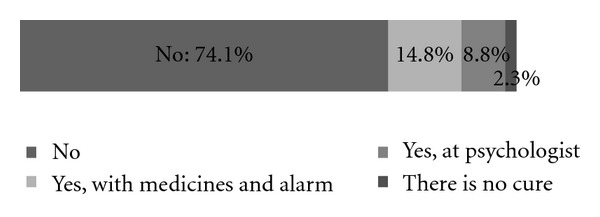
Knowledge of investigated children about treatment of enuresis (Do you know how enuresis can be treated?).

Among incidentally found enuretics very few have been treated (Figure 2). The second questionnaire, carried out in the Nephrology Unit, showed that only 4 children (9.5%) were never treated. The majority among those treated use drugs; the treatment itself lasts on average for about a year. Almost everyone improved their condition. In 25% cases the disorder completely vanished. Types of treatment of enuretics at the Nephrology Unit are shown in Figure 3.  Figure 4 represents knowledge about enuresis treatment among children in elementary schools in Slovenia that was found to be insufficient.

In the second questionnaire we asked enuretics about their quality of life. More than half of them (23, 56%) said that enuresis causes no problems in their lives. The rest of the children were worried that the disorder will never subside (7 children or 17.1%) or that their classmates would find out about their problem (7 children, 17.1%). 18 (40.9%) enuretics admitted that they have tried to hide their problems at least once, 9 of them (21%) have already avoided sleepovers or weekly school trips because of enuresis. 97.1% are of the opinion that they are not being treated differently by their friends, but only 5 (11.9%) stated that their friends know about their condition. Moreover, 22 of them (52.4%) admitted that none of their friends know about their bedwetting problems.  Table 1 shows some quality of life parameters in different life areas. We investigated general well-being of children with PNE, comprehension of themselves, free time, relationships with their family and friends and school activities.

## 4. Discussion

According to the literature we presumed that the PNE would be found in about 10% of children questioned in the survey (Table 2). We found out that it appears in 154 cases (12.4%). Our gained prevalence is comparable to prevalence in France, Netherlands, Turkey, and Korea. Prevalence was determined with questionnaire. We were aware of difficulty of differentiating PNE from other voiding disorders. That is the reason we added questions about daytime incontinence. Due to the subjectivity of questionnaire, there is a possibility of slightly overestimated prevalence. Prevalence of PNE in girls is 11.8% and in boys 13%, but the difference is not statistically significant (*χ* ^2^ = 4.36, *P* = 0.628). Literature implies that there is higher prevalence of PNE among boys which is in accordance with our findings [13].

Our study confirmed the role of genetics in PNE appearance, which has already been confirmed in different studies. PNE is supposedly autosomal dominantly inherited with considerable gene penetrance [2, 3]. Results of our study have confirmed this hypothesis; we learned that there is a statistical difference between the frequency of PNE among relatives of enuretics and nonenuretics. Higher frequency of PNE in different generations of families can also be explained with paying more attention to those kinds of problems. We believe a lot of children are not familiar with bedwetting problems of their relatives, especially their grandparents. Therefore, the frequency might be underestimated.

There have been some reports that the appearance of urge incontinence in patients with enuresis is more frequent. According to Butler et al. it appears in 6.9% of nonenuretics and in 19.5% of enuretics [14]. Our study gave similar results, children mentioned it in 14.4%. A reason for that might also be the fact that we have also included children with an incontinence of urine, caused by waiting too long, as urge incontinence.

It is surprising that despite the fact that PNE is becoming a frequent problem of children and adolescents, we did not find any studies which would show accurate portion of children in general population who are being treated for bedwetting. Results of our study showed that a considerable part of Slovenian children is not treated adequately. The reason for that may be a lack of knowledge of the parents and unsatisfying paediatric treatment at primary health care level. There are rooted opinions among parents about PNE being a psychosomatic disorder which resolves spontaneously and consequently they do not take their children to the doctor. Second questionnaire included children who are treated in the Nephrology Unit. Consequently, the results that 43.2% are treated for bedwetting and 43.2% had already completed the treatment were expected. The majority of those were expectantly treated with medications. The combination of treatment with medications and with alarm brings surprisingly positive results [15], but unfortunately parents in Slovenia have to cover the expenses of treatment with alarm which makes it affordable only for some children. Treatment of PNE is of long duration; most of the children are treated longer than a year (45.5%). Proven success of the treatment is confirmed by data that the treatment has improved the condition in 81.8% of children. 22.7% of those have no bedwetting problem anymore.

According to the answers of children questioned about their willingness to share their experiences and problems with other people, PNE is treated as a taboo subject between youths. Children with PNE are not prepared to talk to coevals about their bedwetting problems because they are afraid to be stigmatized, embarrassed and that their classmates and friends will not accept them. This fear causes a lack of knowledge about PNE among primary school population. Children mostly alleged that they do not know any coevals with the disorder. We tried to define knowledge about PNE in primary school population by checking the familiarity with treatment of mentioned disorder. Results show very evidently that the knowledge about PNE in primary school population is of concern. The reason why children know so little about enuresis may be the fact that 89% children interviewed do not know any coevals who have problems with bedwetting. To our knowledge, there are no data about this in the literature. In addition, the health education might be the problem. There is still a deep-rooted opinion that PNE is a psychological disorder. It is very important and necessary to have better prevention and education in this area. Simultaneously with the study we distributed pamphlets with basic data about PNE and its treatment among all questioned children. We are of opinion that the effective education is still needed in this field, which is one of the important conclusions of the study.

The second questionnaire tried to find out the quality of life of children with PNE. It affects children in all areas of their lives and causes problems which some children face easier and others are more susceptible to them. 22.8% of children questioned frequently face fear of sleep, therefore almost a quarter of them at least occasionally suffer from stress, caused by enuresis. More than a half of them (52.5%) are sometimes in a bad mood when they think of their bedwetting problem, 11.3% are in such condition very often or always. Fear of lifelong bedwetting represents an even bigger problem to 47.7% of questioned children. This data are alarming and we should consider psychological approaches of treatment more often, since children are not capable of facing their problems alone. Although PNE presents a burden in a life of a child, the majority of children (93.1%) think positive and have a positive attitude towards life. Moreover, in spite of the fact that PNE is a disorder which aggravates life of many children and adolescents, we classify it as a disorder which does not have a major influence on the development and course of life. Symptoms of PNE usually appear in homely safe and familiar environment while children are asleep and can immediately get the attention and love from their parents. These facts certainly encourage the child's higher self-esteem since the feeling of being stigmatized is not present in such a large extent and because only the members of the closest family, whom they can trust, are aware of their bedwetting problem.

The occurrence of PNE affects the self-esteem of every child, but in a different manner. Enuretics may have behavioural problems; long lasting disorder can even lead to physiological and psychiatric problems [11]. Results of the questionnaire have shown the presence of shame among children with enuresis (40.9%). The feeling mentioned is especially distinctive at adolescents who feel different from others and even avoid social activities because of that [16]. It is positive that majority (86.4%) of children think that people love them in spite of their bedwetting problem. Reasons for that can be found in powerful influence of parents and the environment. Children feel safe and accepted at home. In other places, where they do not have that feeling of safety, they do not talk about their bedwetting problems. It is likely that they subconsciously filter to whom they are going to trust.

Nowadays, children and adolescents spend their free time differently. For this period of life it is common that they spend a lot of time with their friends because they are looking for acceptance and confirmation from their coevals. According to data published by Warzak, enuretics face different kinds of fears and spend most of their time in society stressed because they are ashamed of their disorder and are trying to hide it [9]. This may be the reason why 34.1% of children questioned spend their free time with their friends and that 56.9% spend most of their free time at home. Symptoms of enuresis usually occur during the night, so 84.1% of questioned children are not impeded from their sport activities.

Despite the fact that the majority of parents accept the occurrence of PNE in their child pretty well, their expectations of their child's self-control and responsibility increase during the child's growing up [11]. Our study has also confirmed that the majority (88.6%) of children get on well with their parents despite of their bedwetting problem and can also talk with them about everything, including bedwetting. Larger part of children also said that their disorder never impedes their family on family trips. However, 25% of enuretics occasionally argue with their parents because of their bedwetting. Moreover, the literature indicates that the negative psychosocial consequences on enuretics are frequent. Enuretics also have a greater risk of becoming victims of emotional and psychological abuse by their family members [9]. In addition, 25% of enuretics said that there is sometimes tension present at home because of their disorder. A quarter of them have a feeling that they burden their parents with the disorder. It is common that relatives know about the state of health of their family members, so it is surprising that only 38.6% of children questioned said their relatives know about their bedwetting problem. PNE is sometimes stigmatized as a shameful disorder. Consequently, 6.8% of asked enuretics said none of their relatives knows about their disorder.

A study by Joinson et al. performed in Great Britain has shown that 24.5% of children with PNE suffer from antisocial distress [17]. One of more important signs of distress among enuretics is avoiding sleeping away from home, with that problem encounter 43.2% of questioned children.

Major part of children (90.9%) do not feel pushed away by their friends as well as they are not teased by them (95.4%). Furthermore, 84.1% of children do not feel impeded by their disorder to make new friendships. However, these facts are not in accordance with the results that 29.5% of them still hide their bedwetting problem from their friends and that 52.3% of them never talk honestly to their friends about their disorder. The reason for this difference is probably the fact that children only talk about their bedwetting problems to the closest friends from whom they do not expect a negative response. They hide their problems from other friends because they are afraid to be labelled. They hide the disorder, not to be treated differently in their environment. 

A study performed by Esposito et al. has shown that enuretics experience learning problems more often than healthy children. The most frequent is the inability to stay focused for a longer period of time; they also have greater problems with learning how to read [12]. Larger part of participants in a survey did not mention learning problems, but 20.5% of them occasionally experience concentration problems as a consequence of night awakenings, 18.2% of them also think that these problems affect their success in school. 

Children with PNE are especially burdened with weekly school trips. 36.3% of them are sometimes anxious about their bedwetting before departure. As already mentioned, enuretics often avoid situations with a great probability of revealing their problems. They are more stressed on weekly school trips than at sleepovers at their friends because they trust their friends more than the whole group of classmates.

## 5. Conclusion

PNE is a common pathology in children; the prevalence in Slovenia is 12.4%. It appears more often among boys and children with genetic predisposition. According to our results, PNE does not lower quality of life of majority of children substantially, although it influences children's lives, especially in their relationships with friends. They often try to hide their problem. Some children also express their distress through lower self-esteem, future strains and lower performance in school. 

PNE is still a rather unknown disorder among Slovenian primary school children. We believe this is also the reason why only a few of these children are being treated. The study also indicates that education of children in this field is still needed.

## Figures and Tables

**Table 1:** Quality of life of Slovenian children with primary nocturnal enuresis.

Quality of life *N* = 44	No answer	Never	Seldom	Sometimes	Often	Always
	*N* (%)					
Common state of health						

I am afraid that I am going to have lifelong wetting problems.	1 (2.3)	22 (50.0)	7 (15.9)	8 (18.2)	3 (6.8)	3 (6.8)
I am often afraid of falling asleep because I might wet my bed.	0 (0.0)	27 (61.4)	7 (15.9)	7 (15.9)	2 (4.5)	1 (2.3)

Comprehension of himself/herself						

I am ashamed because I still wet my bed.	0 (0.0)	26 (59.1)	10 (22.7)	6 (13.6)	2 (4.6)	0 (0.0)

Free time						

I spend most of my free time with my friends.	0 (0.0)	0 (0.0)	3 (6.8)	12 (27.3)	21 (47.7)	8 (18.2)
I spend most of my free time at home.	1 (2.3)	0 (0.0)	6 (13.6)	12 (27.3)	20 (45.4)	5 (11.4)

Relationships in family						

I get on well with my parents although I wet my bed.	0 (0.0)	0 (0.0)	0 (0.0)	1 (2.3)	4 (9.1)	39 (88.6)
I can talk to my parents about everything, including my bed wetting problem.	0 (0.0)	0 (0.0)	2 (4.6)	0 (0.0)	3 (6.8)	39 (88.6)
It seems to me that I am a burden to my parents because of wetting my bed.	1 (2.3)	32 (72.7)	3 (6.8)	7 (15.9)	1 (2.3)	0 (0.0)
My relatives know that I wet my bed.	2 (4.6)	3 (6.8)	12 (27.2)	8 (18.2)	2 (4.6)	17 (38.6)

Relationship with friends						

My friends tease me because I wet my bed.	1 (2.3)	42 (95.4)	1 (2.3)	0 (0.0)	0 (0.0)	0 (0.0)
I hide my bedwetting problems from my friends.	0 (0.0)	15 (34.1)	5 (11.4)	8 (18.2)	3 (6.8)	13 (29.5)
I can talk honestly to my friends about my bedwetting problem.	0 (0.0)	23 (52.3)	7 (15.9)	6 (13.6)	3 (6.8)	5 (11.4)

School						

When I wake up at night because of wetting my bed I have trouble concentrating in school.	1 (2.3)	34 (77.2)	3 (6.8)	4 (9.1)	1 (2.3)	1 (2.3)
Before I go on a school trip I feel very anxious about wetting my bed.	5 (11.4)	23 (52.3)	5 (11.4)	6 (13.6)	2 (4.5)	3 (6.8)

**Table 2:** Prevalence of primary nocturnal enuresis (PNE) in different countries.

Country	Prevalence of PNE	Country	Prevalence of PNE
England [18]	5.8%	The Netherlands [19]	12.1%
Sweden [20]	7.3%	Korea [21]	13.7%
Slovenia [3]	8.7%	Turkey [14]	15.1%
Spain [22]	10.2%	Japan [23]	21.0%
France [24]	11.2%
